# Opportunities and Risks of UK Medical Device Reform

**DOI:** 10.1007/s43441-022-00394-0

**Published:** 2022-04-13

**Authors:** Ji Eun Diana Han, Hussein Ibrahim, Olalekan Lee Aiyegbusi, Xiaoxuan Liu, Eliot Marston, Alastair K. Denniston, Melanie J. Calvert

**Affiliations:** 1 Birmingham Health Partners Centre for Regulatory Science and Innovation, University of Birmingham, Birmingham, UK; 2 Institute of Applied Health Research, College of Medical and Dental Sciences, University of Birmingham, Edgbaston, Birmingham, UK; 3 Centre for Patient Reported Outcomes Research, Institute of Applied Health Research, College of Medical and Dental Sciences, University of Birmingham, Birmingham, UK; 4 Health Data Research UK, London, UK; 5 University Hospitals Birmingham NHS Foundation Trust, Birmingham, UK; 6 Regulatory Horizons Council, London, UK; 7 National Institute for Health Research (NIHR) Birmingham Biomedical Research Centre, University of Birmingham, Birmingham, UK; 8 NIHR Surgical Reconstruction and Microbiology Research Centre University Hospitals Birmingham NHS Foundation Trust, University of Birmingham, Birmingham, UK; 9 National Institute for Health Research (NIHR) Applied Research Collaborative West Midlands, University of Birmingham, Birmingham, UK

**Keywords:** Medical devices, Regulations, In vitro diagnostics, UKCA, CE mark

## Abstract

**Objectives:**

To identify the potential opportunities and risks around future UK regulatory reform of medical devices.

**Design:**

A mixed methods approach, comprising a rapid literature review, one-to-one, semi-structured interviews with key stakeholders, a multidisciplinary stakeholder workshop, and a post-workshop survey.

**Setting:**

United Kingdom.

**Participants:**

32 key stakeholders across the medical device sector were identified both from the public and private sectors.

**Results:**

Opportunities relating to regulatory independence were identified, including the potential to create and implement a regulatory framework that ensures availability of medical devices; innovation and investment potential; and safety to the citizens of the UK. The most significant risks identified included threats to the safety of individual patients and the wider health system arising from the delay in awaiting regulatory approval due to the shortage of approved bodies; and reduced competitiveness of UK market and device manufacturers. Recommendations were identified to mitigate risks, centred on harnessing broader cross-sector collaborations, promoting patient and public partnership, and maximizing international engagement.

**Conclusions:**

The UK’s medical device sector is at a time-critical juncture to construct a regulatory framework to navigate its exit of Europe and respond to Europe's transition to new medical device regulations whilst also addressing the ongoing demand for rapid approval for new devices in response to the global pandemic. Investment, capacity-building, and international engagement will play a central role in mitigating risks and maximizing opportunities for medical device regulation.

**Supplementary Information:**

The online version contains supplementary material available at 10.1007/s43441-022-00394-0.

## Introduction

Medical devices are an essential and increasing part of healthcare delivery. Their applications are hugely diverse, with both hardware (including apparatus, appliances, and instruments) and software (including artificial intelligence as a medical device (AIaMD)) applications [1]. The medical device sector is a highly innovative space, as exemplified during the pandemic when innovators and manufacturers responded with new products ranging from personal protective equipment to in vitro diagnostics to machine learning (ML)-based imaging analytics. This rapid innovation can bring significant societal benefits—directly to individual patients, to population health, and to system efficiencies—but needs to be set within regulatory safeguards that ensure they meet acceptable standards of safety, quality and effectiveness.

Innovation, and regulatory reform to support innovation, is a central part of the UK Government's strategy to respond to the opportunities and challenges arising from leaving the European Union (EU) [2]. As with other sectors, regulatory reform on medical devices can bring new opportunities. It is projected that streamlining the pathway from innovation to market will not only bring benefit to patients sooner, but also accelerate growth in the medical device sector (which currently employs 102,800 people, with an annual turnover of £7.7 billion) [3]. However, medical device companies operate within a global market, and regulatory divergence may result in barriers to the import and export of medical devices, and pose a risk to patients’ health [4].

In preparation for the post-Brexit regime, the UK Government introduced the new UK Conformity Assessed (UKCA; and UKNI for Northern Ireland) mark as a replacement for the existing Conformitè Europëenne (CE) marking system for medical devices being placed on the market in Great Britain [5]. The EU CE mark will continue to be recognised in Great Britain until 30 June 2023, after which all medical devices on the market will require a UKCA mark.

In 2019, the UK government established the Regulatory Horizons Council (RHC), an independent expert committee that identifies the implications of technological innovation, and provides the government with impartial, expert advice on the regulatory reform required to support its rapid and safe introduction. From 2020 to 2021, the RHC undertook a review of the medical device sector culminating in a report submitted to the UK Government in July 2021. As part of the evidence underpinning that report, the RHC commissioned a multi-stakeholder mixed-methods research study which was undertaken by Birmingham Health Partners Centre for Regulatory Science and Innovation. This study engaged a diverse group of stakeholders to explore key questions prioritized by the RHC: First, to identify the potential opportunities and risks around future UK regulatory reform of medical devices; second, to discuss how the UK can encourage international investment, innovation, and improve safety in the medical devices through regulatory and non-regulatory change; third, to determine implications of ending the use of the EU CE mark for medical devices in Great Britain and the mitigations needed to facilitate the move to the UKCA mark; and fourth, to explore potential alternative routes to market for medical devices that are currently being used internationally that could be transposed to the UK market and regulatory system.

## Methods

### Study Design

A mixed methods approach was used to collate multiple sources of evidence and representative stakeholder views. This comprised: (i) a rapid literature review; (ii) one-on-one, semi-structured interviews with stakeholders; (iii) a multidisciplinary stakeholder workshop to review initial findings and discuss areas of agreement and disagreement; and (iv) a post-workshop survey to further explore areas of contention identified during the workshop. An independent advisory committee was appointed comprising representatives from Innovate UK, British In Vitro Diagnostics Association, and the Association of British HealthTech Industries.

This study was approved by the ethical review committee at the University of Birmingham, UK (ERN_20-1852).

### Rapid Literature Review

A rapid literature review was conducted on 08 January 2021. PubMed and Google Scholar were used to search published literature and Google Search Engine was used to search grey literature to find relevant literature under two themes: (i) reported implications of ending the use of the EU CE mark for medical devices in Great Britain and (ii) potential alternative routes to the UK market for medical devices.

Composite and extended terms containing the roots “medical” and “device*” were combined with terms relating to the transition to the UKCA mark, such as “Brexit”, “EU CE” “UKCA” (Online Appendix 1). The first 100 citations from Google Scholar and Google Search Engine were screened. Citations were independently screened by two co-investigators according to pre-defined inclusion and exclusion criteria (Online Appendix 1). Disagreements were resolved via consensus.

### Qualitative Research

#### Semi-structured Interviews

Prospective participants were provided with the electronic participant information sheet and consent form (Online Appendix 2). We used a maximum variation sample of a heterogeneous group of stakeholders, primarily focusing on those involved in the life cycle of a medical device, both from the public and private sectors (including: medical device regulation consultants, representatives from small to medium enterprise or start ups, providers of medical device testing services, academics, clinicians, UK Government bodies, trade association or industry groups, patient and public partners and large enterprise). We approached prospective interviewees from December 2020 to February 2021 by email. Participants were recruited until a point of saturation was reached, i.e. when the additional interviewees provided no further information.

#### Multidisciplinary Stakeholder Workshop

All 30 participants involved in the online interviews and 19 further participants identified via stakeholders’ recommendations, were invited to an online workshop to explore areas of concern or contention highlighted by the literature review and stakeholder interviews. The workshop was conducted via video-conferencing (MS Teams, Microsoft) on 09 February 2021.

#### Post-workshop Survey

A post-workshop anonymized online survey (Online Appendix 3) was distributed via Qualtrics Survey Software between 19 February 2021 and 05 March 2021. The survey was designed to provide workshop attendees with an opportunity to identify any additional concerns that might have arisen since the workshop, and contribute additional feedback.

### Selection of Study Participants

To provide robust, multi-stakeholder, cross-sector input, to inform recommendations for the UK Government around medical devices regulatory reform that maximizes opportunity and minimizes risks, we recruited stakeholders likely to give in-depth information on regulations around medical devices, without any geographical restriction. The project team worked with the independent advisory committee to identify a list of stakeholders to contact and supplemented this list by: (i) searching publicly available listings from the relevant UK Government bodies, industry-specific organizations, and academic institutions, and (ii) snowball sampling from interviewees. Representatives from the UK Medicines and Healthcare products Regulatory Agency (MHRA) were not recruited so as to maintain independence.

### Data Collection

We performed online interviews via video-conferencing (MS Teams, Microsoft) between 04 January 2021 and 02 February 2021. A total of 30 one-on-one, semi-structured interviews were conducted based on an interview guide with open-ended questions on the stakeholders’ assessment of current UK medical device regulatory framework, as well as their expectations and concerns regarding the reforms. We pilot-tested the interview guides with members of the advisory committee to ensure the questions were appropriately framed and addressed all the relevant aspects of the subject (Online Appendix 4).

### Data Analysis

The interview and workshop narratives relevant to our research questions were identified and analyzed thematically using the framework approach [6, 7]. This method allowed a comprehensive review of descriptive narratives that was driven by stakeholders’ original accounts and literature review. The interviews were transcribed, reviewed, and coded independently by JDH and HI, using the stakeholder interview questions as an initial thematic framework. Textual codes were grouped into clusters around similar and interrelated concepts and a matrix of recurrent themes were identified and systemically analysed within Google Sheets. Key statements from interviews and the workshop are reported verbatim to respect the integrity of the statements without any bias. The inductive coding process was reviewed internally by OLA, and any disagreements about the coding were discussed and resolved with third reviewers (MJC, EM, AKD).

## Results

Themes were initially explored in rapid literature reviews where there was pre-existing literature to support this, followed by the qualitative studies; themes which did not have significant pre-existing literature (either by their nature or recency) were explored through the qualitative studies. The breadth of views and typical representative views are reported.

### Rapid Literature Review

The rapid literature review identified a total of 307 non-duplicate articles under the theme*, Key implications of the transition to the UKCA or UKNI mark*. Following title and abstract screening, 119 papers were removed, and 108 articles were reviewed in full text. A full-text screening resulted in the exclusion of 77 articles resulting in a total of 31 articles included in the final review for this theme. Reasons for exclusions were that articles did not relate to medical devices or in vitro diagnostic devices; published before 31 December 2019; or were reporting factual information of the transition to the UKCA mark (Online Appendix 5). A total of 270 non-duplicate titles from studies of any format, were identified for the theme: *Evaluating alternative regulatory frameworks or regulatory components for potential adoption by the UK*. 112 were excluded after title and abstract screening and 158 articles were reviewed in detail. Of these, 135 did not meet the inclusion criteria, specifically the papers did not report on the new, alternative or international routes to the UK market. Overall, 23 articles were included in the final review for this theme (Online Appendix 6).

### Stakeholder Group Representation for Qualitative Studies

A total of 32 key stakeholders across the medical device sector participated in the study (30 in qualitative interviews and 26 in the consensus meeting). Of the 32 participants, 30 were from the UK, one was from mainland Europe (Germany), and one was from the USA. This distribution reflects the UK context of this work (Table 1).

**Table 1:** Stakeholder Groups Represented by Participants (*n* = 32)

Main Role of Participants Within the Medical Device Sector	*N* (%)
Consultancy for regulations around medical devices	6 (19)
Small to Medium Enterprise (SMEs) or medical device start-ups	5 (16)
Providers of medical device testing and certifying services	5 (16)
Academics and clinicians	5 (16)
UK Government-related bodies	5 (16)
Medical device trade association or industry group	3 (9)
Patient and public partners	2 (6)
Large enterprise	1 (3)

### Theme 1: Potential Opportunities and Risks Around Future UK Regulatory Reform of Medical Devices

Based on literature review findings and interview responses, we categorized a range of opportunities and risks around future UK regulatory reform of medical devices into four key areas: (i) patient and public access to high quality medical devices; (ii) international investment and innovation; (iii) patient and user safety; and (iv) global standing in regulation of the life sciences sector (Table 2).

**Table 2:** Key Opportunities Around Future UK Regulatory Reform of Medical Devices

Patient and public access to high quality medical devices
∙ Design efficient, streamlined, UK-specific regulatory processes that ensure high quality, safe, and effective devices are made available on the UK market in a timely manner
International investment and innovation
∙ Make NHS data more accessible to innovators to use for research and development (R&D) of medical devices, especially novel, data-driven devices such as those including artificial intelligence (AI) and machine learning (ML) components ∙ Focus its regulatory resources on complex, cutting-edge medical devices, rather than “run-of-the-mill” ones ∙ Introduce and utilize accelerated regulatory pathways that are similar to Breakthrough Device Designation of the US FDA
Patient and user safety
∙ Involvement of patients and the public as key stakeholders in medical device R&D ∙ Increase the emphasis placed on post-market surveillance (PMS) ∙ Encourage greater collection of patient-centred data such as patient-reported outcomes ∙ Foster a culture of learning, rather than a culture of blame, from patient safety incidents
Global standing in regulation of life sciences sector
∙ Maximise new and existing international collaborations ∙ Promote harmonization with the US, Commonwealth countries, individual EU member states, and elsewhere

#### Patient and Public Access to High Quality Medical Devices

Overall, stakeholders believed that the UK regulatory reforms present a timely opportunity to enhance patient and public access to high-quality and safe medical devices by streamlining the regulatory approval process that aligns with the interest of the UK public. A representative view was that “there is too much noise (in the European Committee), as regulations need to work and be relevant to 27 different member states and their national health strategies”. Their view was that a UK-oriented regulatory system could produce the best outcomes for the UK National Health Service (NHS), life sciences sector and internal market. However, many stakeholders highlighted that regulatory divergence from the international regulatory framework is likely to increase the burden on the regulatory authorities and device companies. Participants were concerned that this, in turn, would increase the delay and cost in placing the devices in the UK market, and would ultimately decrease the availability of medical devices for UK patients.

#### International Investment and Innovation

With regards to potential opportunities to accelerate the adoption of cutting-edge technology and ensure innovation in the UK, stakeholders expressed health data as the “most obvious and the strongest asset, provided by the NHS”. Respondents, mainly from the small to medium enterprises (SMEs), were of the opinion that by leveraging the development of data-driven devices and enhancing scalability of collaboration with NHSx, NHS Digital (organizations now absorbed into NHS England and NHS Improvement), and other organizations responsible for the digital services delivery, the UK will foster a globally competitive environment for the medical device start-ups. One common view was that the UK should prioritize its regulatory resources and implement a specialist regulatory system to safely support the creation of the more innovative and complex medical devices over “run-of-the-mill” devices to secure its competitiveness in the global market.

#### Patient and User Safety

Participants across all stakeholder groups agreed on the need for greater involvement of public and patient advocates in regulatory activities across the lifetime of a medical device. Stakeholders suggested several ways to promote patient and user safety, including the use of patient-centred clinical data to demonstrate device compliance, a stronger vigilance, and post-market surveillance process, coupled with a publicly funded and accessible database on medical devices. Stakeholders raised concern over implementation of a more relaxed regulation relative to the current regulation. The prevailing concerns over such regulatory “shortcuts” were of compromising the overall quality of medical devices, which, in turn, may incur considerable societal cost, as well as potential harm to patient safety.

A widely shared opinion suggested that there is an opportunity for the new UK regulatory system to “be a ‘halfway house’ between EU Medical Device Directive 93/42/EEC (MDD) and Medical Device Regulation 2017/745 (MDR) that brings in safety aspects of MDR but keeps the lighter touch of MDD”, to encourage a system that is efficient enough for investors and innovators to use it, whilst safe enough to protect patients and users.

#### Global Standing in Regulation of Life Sciences Sector

Stakeholders, regardless of their role and experience, agreed on maximising both new and existing collaboration with international standards and other international regulatory regimes, to identify where the UK’s domestic regulatory system could be made more streamlined and effective. The concept of regulatory harmonisation with wider international standards, including the US, Commonwealth countries, individual EU member states, and elsewhere, was also highly valued, and was suggested as a key element for the UK “to preserve the leading role in shaping global regulatory sciences”.

### Theme 2: Regulatory and Non-regulatory Changes in the UK to Encourage International Investment, Innovation, and Improve Patient Safety

Stakeholders were invited to identify the regulatory and non-regulatory changes in the UK that would improve international investment, innovation, and patient safety (Table 3).

**Table 3:** Measures to Enable the UK Encourage International Investment, Innovation, and Patient Safety in the Medical Device Sector

How can the UK encourage international investment in the medical device area?
*Regulatory changes* ∙ Ensure that new UK regulations are sufficiently aligned with international regulations ∙ Encourage early engagement with and support for companies developing high-risk medical devices *Non-regulatory changes* ∙ Provide clear guidance on new regulations ∙ Utilize investment incentives ∙ Optimize NHS procurement process ∙ Facilitate access to NHS data and infrastructure ∙ Strengthen international R&D collaboration
How can the UK encourage innovation in the medical device area?
*Regulatory changes* ∙ Coordinate the clinical evidence requirements for regulatory approval and health technology assessment ∙ Focus innovation on clinical need using target product profiles and horizon scanning ∙ Introduce alternative routes to market for innovative and breakthrough devices *Non-regulatory changes* ∙ Provide clear guidance regarding regulatory routes to market ∙ Continue to provide financial incentives for medical device R&D ∙ Strengthen collaborative partnership between industry and the NHS ∙ Invest in translational and regulatory sciences
How can the UK improve safety in the medical device area?
*Regulatory changes* ∙ Increase the emphasis placed on post-market surveillance and improve post-market surveillance processes ∙ Use medical device databases and registries and unique device identifiers ∙ Introduce a post-approval ‘transition’ phase to the regulatory route to market before routine clinical use *Non-regulatory changes* ∙ Promote patient and public involvement and use of patient-reported outcome measures ∙ Encourage voluntary reporting of suspected medical device incidents by patients, the public, and healthcare professionals ∙ Foster a culture of learning rather than a culture of blame

#### How Can the UK Encourage International Investment in the Medical Device Area?

Stakeholders regarded alignment with international regulatory authorities as desirable, and a key component of attracting international investment in the medical devices area. A typical view was “it is important for the (UK) regulatory processes to open up more than one market” with an emphasis from all stakeholder groups on encouraging greater access to international markets. The following ways of increasing entry to the international market were proposed: aligning the UK conformity assessment with the EU CE; optimising global regulatory harmonisation; and agreeing on a mutual recognition with other countries (the Commonwealth countries and the US were particularly highlighted). One stakeholder critically noted that a regulatory ecosystem that encourages and supports companies specialising in high-risk devices from the earliest stage, would lower the risk of nonconformance, offer investor confidence and, thereby, drive international investment in the industry.

Participants also provided several non-regulatory measures for the purpose of encouraging investment. These included but were not limited to: (i) a greater clarity around new regulations; (ii) investment incentives, such as grants, tax credits, and subsidized manufacturing infrastructures; (iii) optimization of the NHS data access and procurement process; (iv) international collaborations between academia and industry. Such elements were commonly identified as non-regulatory practice to encourage innovation.

#### How Can the UK Encourage Innovation in the Medical Device Area?

With reference to promoting innovation, stakeholders expressed the need to decrease the burden involved in acquisition of clinical evidence for regulatory approval and health technology assessment (HTA) decisions. Some interviews centred on the debate on guiding developers to understand the end-user needs in the context of providing target product profile and publicly funded horizon scanning. Furthermore, respondents promoted the idea of adopting a regulatory pathway similar to the Humanitarian Device Exemption and Breakthrough Device Designation of the US FDA to provide incentives for development of devices for the management of rare or life-threatening conditions; it should be noted that the MHRA do in fact have an exceptional use route on humanitarian grounds.

#### How Can the UK Improve Safety in the Medical Device Area?

Regarding improvement in patient safety, the area most strongly highlighted was the need to improve post-market surveillance (PMS). Respondents suggested that systematic monitoring and analysis of user complaints may provide early warning of possible device-related harms. Another suggestion to enhance PMS was to introduce a post-approval transition period to gather further evidence on safety before deploying and scaling up for routine clinical use. Additionally, a publicly accessible, national database of registered medical devices using unique device identifiers to “promote transparency around the evidence for effectiveness and safety of medical devices” was proposed.

The majority of the respondents maintained that patient and public involvement in the design and development of clinical trials of medical devices would best capture and address users’ needs and enhance safety. Representatives from patient and public partners highlighted the growing recognition of validated patient-reported outcomes as a means of involving “patients in a meaningful manner.”

### Theme 3: Key Implications of the Transition to the UKCA or UKNI Mark

Stakeholders identified that the end to the use of the EU CE mark for medical devices in the UK and the move to the UKCA mark (or UKNI mark) pose unique implications for different stakeholder groups. Implications and mitigations were considered according to: (i) those most relevant to regulators, (ii) those most relevant to medical device companies; (iii) and those most relevant to patients and the public (Table 4).

**Table 4:** Key Implications and Mitigation Works for the Move to the Use of UKCA Mark in Medical Devices Placed in GB from 1 July 2023

Key Implications	Mitigation
*Most relevant to regulators* ∙ Surge in demand for the services of UK Conformity Assessment Bodies (UK-CABs) in excess of current regulatory capacity ∙ Decrease in the UK’s international regulatory influence	*Most relevant to regulators* ∙ Increase the number of UK-CABs ∙ Prioritize allocation of limited UK regulatory resources ∙ Encourage UK-CABs to expand their coverage of high-risk medical devices ∙ Expand the MHRA’s role and responsibilities ∙ Encourage EU-based Notified Bodies (NBs) to perform UKCA and EU CE conformity assessment in parallel in anticipation of a significant degree of overlap in requirements for attestation by both bodies ∙ Increase coordination across regulators, health technology assessors, and procurers
*Most relevant to medical device manufacturers* ∙ Increase in costs to medical device companies due to dual regulatory burden ∙ Unequal impact on small vs. large medical device companies ∙ Reduction in number of medical device companies prioritizing UK market authorisation ∙ Decrease in the amount of UK-based medical device research	*Most relevant to medical device manufacturers* ∙ Provide medical device companies with clear, transparent, and unified guidance ∙ Encourage mutual recognition of clinical evidence across UK, EU, and US regulatory systems ∙ Incentivise medical device companies to develop and sell devices in the UK ∙ Develop state-of-the-art regulation for complex and innovative medical devices to attract innovators and investors ∙ Extend the transition period for all or some medical devices
*Most relevant to patients and the public* ∙ Reduction in availability and choice of medical devices ∙ Unequal impact on patients with rare conditions, compared to common conditions ∙ Confusion and anxiety amongst medical device users	*Most relevant to patients and the public* ∙ Provide patients and the public with clear, transparent, and understandable information

Of 26 workshop attendees, a total of 16 stakeholders completed the anonymized post-workshop survey. 56.2% (9/16) of respondents said that the medical device industry is able to meet the new UKCA mark requirements by the deadline of 01 July 2023. Respondents held contrasting opinions over the degree of continued regulatory alignment in the current requirements for the UKCA mark with the EU CE mark requirements. Whilst some assumed that there would be a high degree of similarity between the two requirements that should minimize the regulatory burden on device manufactures, others criticized the potential disparity and the delay in providing the full details of the UKCA requirements. Stakeholders also expressed concerns that this deadline can only be met with “practical support from the Government” and said that it was essential that “any changes made to the UK regulations are agreed and communicated by the end of 2021 at the latest”. Additionally, half the survey respondents (8/16) strongly agreed that the UKCA deadline of 01 July 2023 poses a potential risk to being able to provide new and existing devices to the patients in the UK (Online Appendix 7).

### Theme 4: Evaluating Alternative Regulatory Frameworks or Regulatory Components for Potential Adoption by the UK

Finally, three regulatory systems in operation internationally were identified as particularly relevant to the regulation of medical devices in the UK. This included the Medical Device Single Audit Programme (MDSAP) [8], U.S. Food and Drug Administration (FDA), and Mutual Recognition Agreements (MRAs). Provided that these systems are not mutually exclusive, stakeholders proposed the idea of the UK choosing to adopt certain aspects or regulatory stages of each system.

Participants suggested that joining the MDSAP as a Participating Country would reduce the regulatory burden and facilitate a more efficient route to international markets, by satisfying quality management system (QMS) requirements across multiple authorities. With regard to MDSAP, MHRA is currently an Official Observer and is considering becoming a member. The main area of criticism in this regard was inadequate coverage of QMS requirements over the entire regulatory approval process. Concerns were expressed that additional QMS activities and harmonization of technical documentation, especially around Software as a Medical Device (SaMD), may be required to fulfil the regulatory requirements of specific participating jurisdictions.

Implementation of a more extensive equivalence-based approval process that is similar to 510(k) of US FDA were suggested as an alternative route to increase the speed, number, and the diversity of devices made available in the UK. The 510(k) pathway’s efficiency is achieved by granting regulatory clearance from low-to-moderate risk devices through demonstration of substantial ‘equivalence’ to a legally marketed predicate device.

In contrast, some stakeholders expressed the fear that the lack of clear consensus on how much divergence is permitted before a device is no longer equivalent to its predicate may result in negative implications on patient safety.

Stakeholder engagement identified that establishing MRAs in conformity assessment certificates of medical devices will address the anticipated capacity gap in UK registration, which can promote efficiency in both the UK’s regulatory system and the regulatory systems of its international counterparts. It was highlighted that this would allow MHRA to a proportionate allocation of regulatory resources towards medical devices with potentially higher public health risk or those with a higher public interest. However, industry is concerned about the further delay involved in building the trust that is required to negotiate bilateral or multilateral MRAs.

## Discussion

This mixed methods study of stakeholder views provides a series of important insights into the potential diverse impacts of future UK regulatory reform in the medical device sector. Analysis of the literature review, semi-structured interviews, workshop, and post-workshop survey identified a number of major themes in which UK regulatory reforms are seen as a balance between opportunities of regulatory independence and the risk of regulatory divergence.

Theme 1 addressed the opportunity and risk of the current and evolving scenario, specifically the opportunities and risk around future UK regulatory reform in the medical device sector. In response, stakeholder insights highlighted the following priority areas: (i) patient and public access to high quality medical devices; (ii) international investment and innovation; (iii) patient and user safety; and (iv) global standing in regulation of the life sciences sector.

Theme 2 invited stakeholders to use their expertise and experience to address these opportunities and risks, specifically to consider the regulatory and non-regulatory changes in the UK to encourage international investment, innovation, and improve patient safety. This elicited a broad range of suggestions but with recurrent themes including: clarity and certainty (the regulatory requirements are known, well-communicated, and any change is provided with due warning); alignment or mutual recognition (reducing friction in export and import), streamlining (e.g. through regulation into health technology assessment and through to procurement), and efficient, digitally enhanced safety monitoring (to improve safety, reduce burden, and potentially enable earlier deployment).

Theme 3 highlighted specific considerations related to the transition to the UKCA and UKNI marks and noted that the risks and mitigations are distinct for regulators, manufacturers, and the patients and public.

**Fig. 1.**
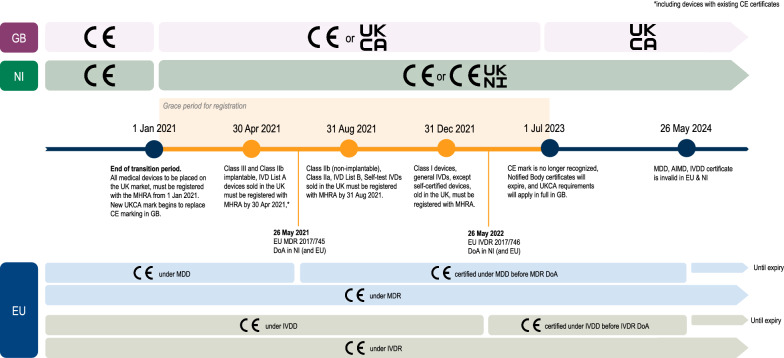
Timelines for Post-Brexit UK legislation for medical devices, in relation to EU Medical Device Regulations 2017/745 (MDR) and in vitro Diagnostic Medical Device Regulations 2017/746 (IVDR)

Theme 4 highlighted opportunities to learn from or align to examples of best practice internationally. There was broad consensus that there is value in participating in international initiatives such as MDSAP and seeking to reduce friction in import and export through alignment or mutual recognition agreements (Fig. 1).

The findings of our study need to be set in the context of the wider, rapidly evolving environment in which the UK’s medical devices sector sits. There are a number of major drivers that shape this environment, many of which are dynamic, and some of which may change rapidly. Two recent and now immutable agents of change for the medical device sector are: first, the transition by the EU from Directives (Medical Device Directive [9], In Vitro Diagnostic Directive [10] and the Active Implantable Medical Device Directive [11]) to the Regulations (Medical Device Regulation (MDR) [12] and In Vitro Diagnostic Regulation (IVDR) [13]; and second, the choice by the UK to leave the EU and establish regulatory independence (including the UK CA mark and the UKNI mark) [14]. This regulatory independence leads to the much-increased influence of a third factor: UK government policy and its potential effect on UK regulation. Previously UK regulatory policy had to align to wider EU decisions and any change was cautious and highly sign-posted [15]. This brought advantages to the device sector of relative certainty and longer-term stability, but disadvantages of poor responsiveness to new challenges, and an inability to tune regulation to the opportunities and requirements of the UK. In contrast, the UK government could now bring sweeping changes to the regulatory framework as it seeks to strongly pursue its own agenda, which is currently strongly pro-innovation.

In February 2021, the UK Government commissioned a Taskforce on Innovation Growth and Regulatory Reform (TIGRR) to “identify and develop proposals across a range of areas that will drive innovation, growth and competitiveness through regulatory reform. ” [16] This reported to the UK government on 16 June 2021, and included a number of specific recommendations relevant to the UK medical devices sector. The view of the reports was that “In establishing our own regulatory framework the UK can now set our own Innovative Medical Devices regime to support this growing sector and anticipate the growing use of software and AI in medical devices. A framework of regulated digital products and devices needs a robust quality system for data management as part of the approval. This could be supplemented with some form of post-marketing surveillance (PMS) as one would see with traditional regulated medical devices.” The report was welcomed by the Prime Minister, and provides an indicator of the UK’s current direction of travel even if not all the specific recommendations survive the journey from report to policy to action.

Our study noted a number of themes that align with the findings and recommendations of the TIGRR report [16], as well as the more recent Life Science Vision for the UK [17], notably to build on some of the unique strengths of the NHS and the opportunity to more effectively use NHS data (including through greater centralisation). Unlike the TIGRR report, our study provides qualitative evidence from diverse stakeholders that recognizes some of the challenges that need to be overcome including current fragmentation into data silos, non-interoperability between datasets [18], and ambiguous data ownership [19] that exist within the current health and legal system. As highlighted in the TIGRR report and echoed by the participants in our study, leveraging existing assets and setting out a “new ambitious regulatory framework”, including approaches such as the Accelerated Access Pathway, will enable a timely response to innovative technology, including AI and ML components. This, alongside a more proportionate, targeted system-based approach, would offer the UK improved capacity and the flexibility to rapidly adapt to the future advancement of digital technologies. Aspirations such as these will require collaborative partnership across the industry and the UK Government.

An important balance to the emphasis on innovation and growth of the TIGRR report, is the Independent Medicines and Medical Devices Safety Review (IMMDSR) chaired by Baroness Cumberlege [20]. This report puts the emphasis firmly on patient safety, an important theme arising from our study. The IMMDSR also highlights the contribution that greater patient and public involvement can make to this. As with our study and the TIGRR report, it highlights the potential for more effective use of NHS data to detect safety issues early and to ensure that all parts of the health system including regulatory components are joined up. In line with our study and the TIGRR report, it also highlights the value of patient-reported outcomes and indeed the potential for collection to be enhanced through digital applications. The inclusion of meaningful, measurable data that is directly relevant to end-users, such as patient-reported outcomes, would maximize the chances that all important safety issues are captured during clinical investigation of devices.

One important difference between the findings of our study and the TIGRR report, is around international engagement. The TIGRR report is focused on UK leadership on the global stage. For our participants, this opportunity was recognized, but even higher priorities for international engagement were avoiding import/export friction through alignment (or mutual recognition), adoption of international technical specifications wherever possible, and ensuring that patient safety was not compromised through lower standards; leadership was considered desirable particularly in those areas where no existing models were considered adequate (such as AIaMD). Nationally, close collaboration from different sectors of the system and stakeholder groups can build synergy and accelerate the future of innovation in the UK [21]. This effort, coupled with global partnership and leadership has the potential to better understand which markets or innovation spaces are most vital and helpful to the UK, and ultimately pursue the goal of developing a robust regulatory regime for medical devices that prioritizes patient safety.

To our knowledge, this is the first published study to have assessed the in-depth stakeholder perspectives around the UK regulatory reforms for medical devices. Our findings are based on the recruitment of a diverse set of stakeholder groups representative of those involved in medical device sectors. The study shares limitations of qualitative approaches in general, principally the non-generalizability of study findings. However, the qualitative approach focusing on the depth was suitable for our study since it enabled us to explore, describe, and analyze sensitive issues related to the new regulatory reforms in the UK medical device sector. Though we interviewed a wide range of participants across eight different stakeholder groups, the small number of respondents in some categories made comparison across groups difficult. The interviews and workshop were undertaken during the Covid-19 pandemic, and this may have influenced responses from some individuals through ‘recency bias’, however given that learning from pandemic is an important, global, and ongoing phenomenon we would argue that this enhances rather than reduces the validity of our findings.

Our study highlights that we are at a critical juncture in the life of the UK’s medical device sector. Already responding to the requirements of transitioning from the EU directives to regulations, the medical device sector is challenged by the rapid pivot to an emerging UK-specific regulatory framework under the Medicines and Medical Devices Act 2021 [22], whilst also navigating a global pandemic. Our study provides additional evidence that stakeholders in the medical device sector recognize the significant opportunities that this brings, but also are concerned about the risks to patients and to the industry arising from the current transition, particularly over potential regulatory divergence. They call for a range of mitigations including investment, capacity-building, and international partnership which would enable the UK device industry to be supported and indeed strengthened, and to ensure that patients can still be provided with the medical devices they need.

## Supplementary Information

Below is the link to the electronic supplementary material.

Supplementary file1 (DOCX 425 kb)
