# Supplementary material for: Opportunities and Risks of UK Medical Device Reform
Source: Ther Innov Regul Sci. 2022 Apr 13;56(4):596–606. doi: 10.1007/s43441-022-00394-0 (PMC9007047; doi:10.1007/s43441-022-00394-0)

# Appendix 1. Search terms and Inclusion/Exclusion Criteria for Rapid Literature Review

## Implications of the end to the use of the EU CE mark for medical devices in Great Britain and mitigation work could take place to facilitate the move to the UKCA mark

| **Pubmed** | | |
| --- | --- | --- |
|  | Search Terms | Record no. |
| 1 | medical device |  |
| 2 | medical devices |  |
| 3 | OR (1-2) | 1,561,268 |
| 4 | CE mark |  |
| 5 | CE marked |  |
| 6 | CE marks |  |
| 7 | CE marking |  |
| 8 | conformity europeenee |  |
| 9 | european conformity |  |
| 10 | declaration of conformity |  |
| 11 | UKCA |  |
| 12 | UK CA |  |
| 13 | OR (4-12) | 1,576 |
| 14 | legislation |  |
| 15 | legislations |  |
| 16 | conformity |  |
| 17 | regulation |  |
| 18 | regulations |  |
| 19 | OR (14-18) | 3,384,232 |
| 20 | 3 AND 13 AND 19 | 108 |

| **Google Scholar and Google Search Engine** |
| --- |
| EU CE marked Medical device Brexit UK impact OR impacts OR implication OR implications |

| **Inclusion** | **Exclusion** |
| --- | --- |
| English language | Non-English language |
| Any format of document |  |
| ≥2010 |  |
| Medical devices and/or in vitro medical devices |  |
| Conformity assessment, EU CE mark, UK CA mark |  |
| Debates, discussions, lessons learned, opinions, reflections, and reviews about application of in vitro devices regulation | Factual information about application of in vitro devices regulation |

## Potential alternative routes to market for medical devices that are currently being used internationally that could be transposed to the UK market and regulatory system

| **Pubmed** | | | | |
| --- | --- | --- | --- | --- |
|  | Search Terms | Record no. |  |  |
| 1 | Medical device |  |  |  |
| 2 | Medical devices |  |  |  |
| 3 | OR (1-2) | 1,561,169 |  |  |
| 4 | United Kingdom |  |  |  |
| 5 | Brexit |  |  |  |
| 6 | OR (4-5) | 907,740 |  |  |
| 7 | Healthcare market |  |  |  |
| 8 | Healthcare markets |  |  |  |
| 9 | Health care market |  |  |  |
| 10 | Health care markets |  |  |  |
| 11 | Healthcare sector |  |  |  |
| 12 | Health care sector |  |  |  |
| 13 | Healthcare industry |  |  |  |
| 14 | Health care industry |  |  |  |
| 15 | Healthcare industries |  |  |  |
| 16 | Health care industries |  |  |  |
| 17 | OR (7-16) | 72,186 |  |  |
| 18 | Pre market requirement |  |  |  |
| 19 | Premarket requirement |  |  |  |
| 20 | Device approval |  |  |  |
| 21 | Devices approval |  |  |  |
| 22 | Medical device approval |  |  |  |
| 23 | Medical devices approval |  |  |  |
| 24 | Device approval process |  |  |  |
| 25 | Devices approval process |  |  |  |
| 26 | Medical device approval process | | |  |
| 27 | Medical devices approval process | | |  |
| 28 | Regulatory framework |  | |  |
| 29 | Regulatory science |  | |  |
| 30 | Medical device legislation |  | |  |
| 31 | Medical devices legislation |  | |  |
| 32 | OR (18-31) | 171,996 | |  |
| 33 | 3 AND 6 AND 17 AND 32 | 74 | |  |

| **Google Scholar and Google Search Engine** |
| --- |
| routes to UK market for medical devices OR medical device |

| **Inclusion** | **Exclusion** |
| --- | --- |
| English language | Non-English language |
| Any format of document |  |
| Any date |  |
| Medical devices and/or in vitro medical devices |  |
| Entry to UK market, routes to UK market |  |
| National and international regulations |  |

# Appendix 2. Participant Information Sheet and Consent Form


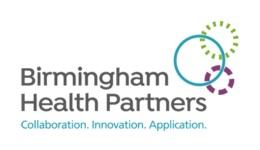


**PARTICIPANT INFORMATION SHEET**

***WHAT IS THE PURPOSE OF THIS STUDY?***

One of the high-profile, immediate healthcare opportunities afforded by the UK’s decision to leave the EU is reform of regulation around medical devices. Post-Brexit regulatory divergence creates opportunities for both health and economic benefits but also risks. There is an urgent need for robust, multi-stakeholder, cross-sector recommendations around future medical devices regulation that maximise opportunities and minimise risks. The purpose of this project is to produce such recommendations for the UK Government.

***WHO IS ORGANISING AND FUNDING THE RESEARCH?***

The Regulatory Horizons Council - an independent committee that identifies the implications of technological innovation and provides the UK Government with impartial and expert advice on the regulatory reform required to support its rapid and safe introduction - has asked the University of Birmingham to lead this research. The project is funded by a Research England Quality-Related Research (QR) Strategic Priorities Fund.

***WHY ARE WE APPROACHING YOU?***

We are inviting individuals who have expertise in medical devices and/or the regulation of medical devices. Your participation will help us to develop recommendations around future medical devices regulation for the UK Government.

***WHAT WILL HAPPEN TO ME IF I TAKE PART?***

Interview

You will be invited to take part in a semi-structured interview with a member of the research team.

The aim of the interview is to identify and prioritise recommendations for future regulatory reform around medical devices. We are hoping to discuss the following items: (a) lessons learnt from Covid-19 regarding in-vitro medical devices regulations; (b) potential alternative routes to the UK market for medical devices; and (c) the implications of a potential hard stop of EU CE marked medical devices. We are happy to be guided by your knowledge/experience and focus the interview on the item(s) that you feel most comfortable/able to discuss.

The interview will be conducted online via video call or by phone. The interview will last approximately 40 minutes. The date and time of the interview will be agreed between you and the research team. All interviews will be conducted between **11 January 2021 – 02 February 2021**, in advance of the consensus meeting on 09 February 2021.

You can choose to answer the questions in as little or as much detail as you feel comfortable. Feel free to ask to skip questions or ask to return to questions later in the conversation.

Please note that the interview will be recorded. The recording will be used to generate an anonymised transcript to facilitate subsequent analysis and then be destroyed. The anonymised transcripts will be securely stored on a password-protected encrypted University server and will not be shared outside the research team.

Consensus meeting

You will be invited to take part in a consensus meeting with members of the research team and other experts like yourself.

The aim of the consensus meeting is to debate and reach consensus on a set of recommendations for future regulatory reform around medical devices. Areas of agreement and contentious issues identified after an analysis of interviews will be discussed. A voting system will be used and results will be stored in an anonymised format.

The consensus meeting will be conducted online via video call on 09 February 2021 (TBC). Ideally, you would attend both the interview and the consensus meeting. However, if you are unable to take part in an interview, that does not preclude you from taking part in the consensus meeting.

Please note that the consensus meeting will be recorded. The recording will be used to generate an anonymised transcript to facilitate subsequent analysis and then be destroyed. The anonymised transcripts will be securely stored on a password-protected encrypted University server and will not be shared outside the research team.

***WHAT WILL HAPPEN IF I DON’T WANT TO CARRY ON WITH THE STUDY?***

Interview

You may withdraw permission for us to use your information without giving a reason up to 5 working days after the interview. This period is considered to be enough for you to decide whether you still want to be part of the study and for the research team to prepare the collected information for analysis. After this point, the information will be integrated into the analysis and it will not be possible to disaggregate it. You should email the study researchers Dr Hussein Ibrahim ([hussein.ibrahim@doctors.org.uk](mailto:hussein.ibrahim@doctors.org.uk)) and/or Dr Diana Han ([d.han@bham.ac.uk](mailto:d.han@bham.ac.uk)) if you wish to withdraw. Once the withdrawal email has been received all the data and information you provided will be deleted.

Consensus meeting

You can choose to not attend the consensus meeting but it will not be possible to disaggregate your data from the consensus process. If you decide to withdraw after the consensus meeting, the data will not be able to be disaggregated and removed from the meeting.

***WHAT WILL HAPPEN TO THE RESULTS OF THE RESEARCH STUDY?***

We aim to publish the results of this research in relevant scientific journals. You will not be identified in any publication(s). We can keep you informed on any publication(s) arising from this study if you wish. Simply complete the relevant section in the consent form.

***WHAT IF THERE IS A PROBLEM?***

This study has a low risk of physical or significant psychological harm. If you wish to complain or have any concerns about any aspect of the way you have been approached or are treated during this study, you can contact the principal investigator, Prof Melanie Calvert.

[Phone:](about:blank) +44 (0) 1214 148 595

Email: [m.calvert@bham.ac.uk](mailto:m.calvert@bham.ac.uk)

***WILL MY PARTICIPATION IN THIS STUDY BE KEPT CONFIDENTIAL?***

Yes, all information that is collected about you during the course of the research will be anonymised and kept confidential.

***WHO HAS REVIEWED THE STUDY?***

The study was approved on 15 December 2020 by the University of Birmingham Ethics Review Committee, Ethical Review ERN_20-1852.

***WHAT IF I HAVE ANY COMMENTS?***

If you have a concern about any aspect of this study you should ask to speak to the study lead researchers Dr Hussein Ibrahim ([hussein.ibrahim@doctors.org.uk](mailto:hussein.ibrahim@doctors.org.uk)) and/or Dr Diana Han ([d.han@bham.ac.uk](mailto:d.han@bham.ac.uk)). Alternatively, the principal investigators, Prof Melanie Calvert (+44 (0) 1214 148 595 or [m.calvert@bham.ac.uk](mailto:m.calvert@bham.ac.uk)) or Dr Eliot Marston (+44 (0) 1214 149 020 or [e.d.marston@bham.ac.uk](mailto:e.d.marston@bham.ac.uk)) will do their best to answer your questions.


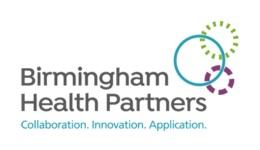


**CONSENT FORM**

Please indicate whether you agree or not and initial each section

| 1. I confirm that **I have read** and **understood the participant information sheet** for the above study and have been given the opportunity **to ask questions** and have these answered satisfactorily. | Agree/do not agree |
| --- | --- |
| 1. PARTICIPATION |  |
| 2a. I agree to **take part in the individual online video or phone interview** and consent to the meeting to **be audio recorded**. I agree that **any of the words** **I may use** during the meeting can **be used anonymously** in the presentation of the research. | Agree/do not agree |
| 2b. I agree to **take part in the online consensus meeting (09 February 2021)** and consent to the meeting to **be audio recorded**. I agree that **any of the words** **I may use** during the meeting can **be used anonymously** in the presentation of the research. | Agree/do not agree |
| 1. RIGHT TO WITHDRAW   I understand that my participation is **voluntary** and that I am **free to** **withdraw** any time prior to the interview and/or consensus meeting without giving a reason. However, I understand that if I **withdraw after the interview and/or consensus meeting** has taken place my anonymised **data may still be used.** I am aware of who I contact should I wish to request a withdrawal. | Agree/do not agree |
| 1. PRIVACY & CONFIDENTIALITY |  |
| 4a. I understand that the **audio recordings** of the interview and/or consensus meeting will be destroyed immediately following transcription. I give my permission for other authenticated researchers to have access to the **anonymised transcription.** | Agree/do not agree |
| 4b. I understand that all personal information will remain **confidential** and that all efforts will be made to ensure I cannot be identified. | Agree/do not agree |
| 4c. I understand that my data gathered in this study will be stored **securely, safely and in accordance** with the Data Protection Act (2018). | Agree/do not agree |
| 1. DISSEMINATION |  |
| 5a. I wish to be acknowledged as a contributor in any subsequent publication(s) or literature. | Agree/do not agree |
| 5b. I wish to receive a copy of any subsequent publication(s) or literature. | Agree/do not agree |
| 1. I agree to **my** **email** being used in any circulation email discussions **with** **other consensus panel members.** | Agree/do not agree |

Name of participant (please print): ________________________________

Signature: ___________________________ Date: __________________

# Appendix 3. Post-workshop Online Questionnaire


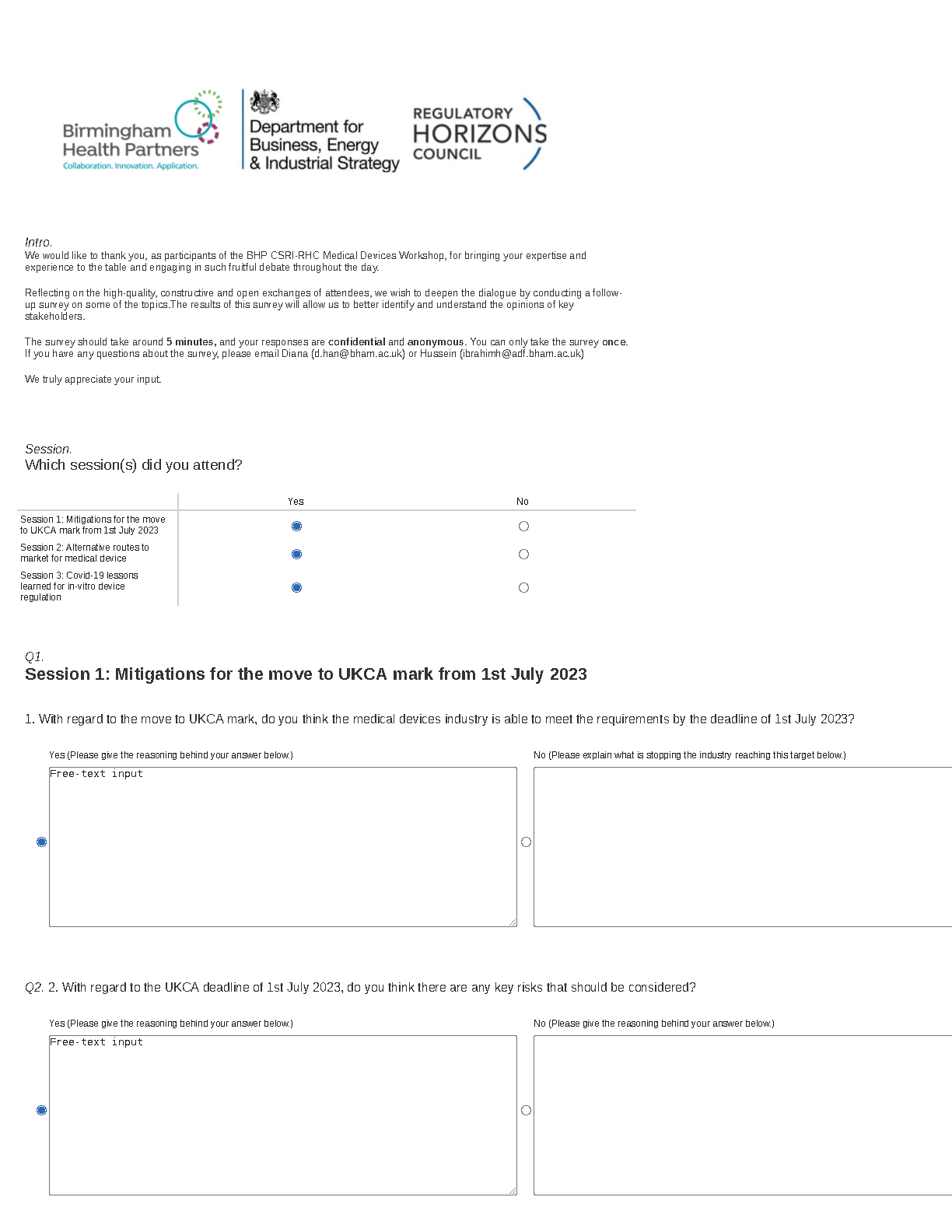

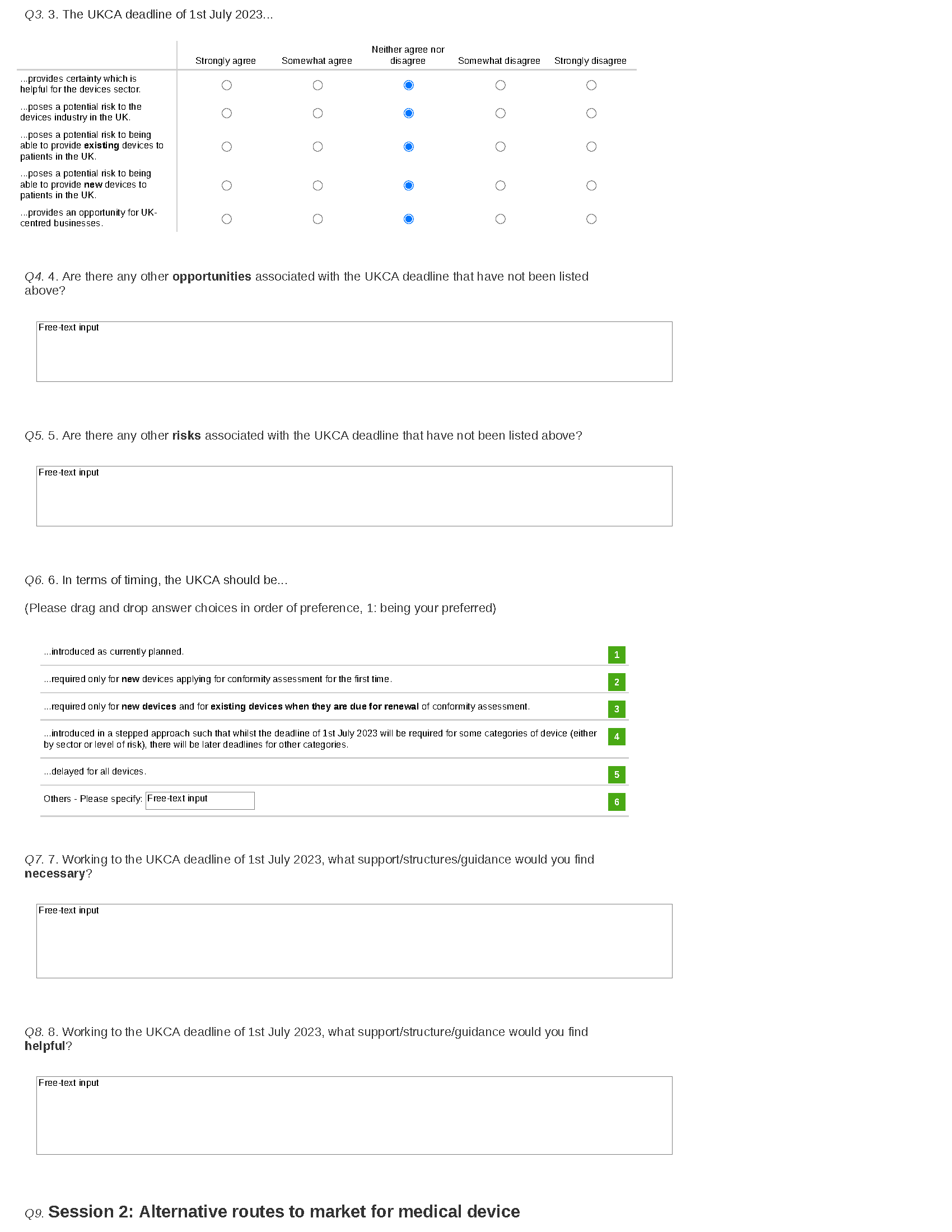

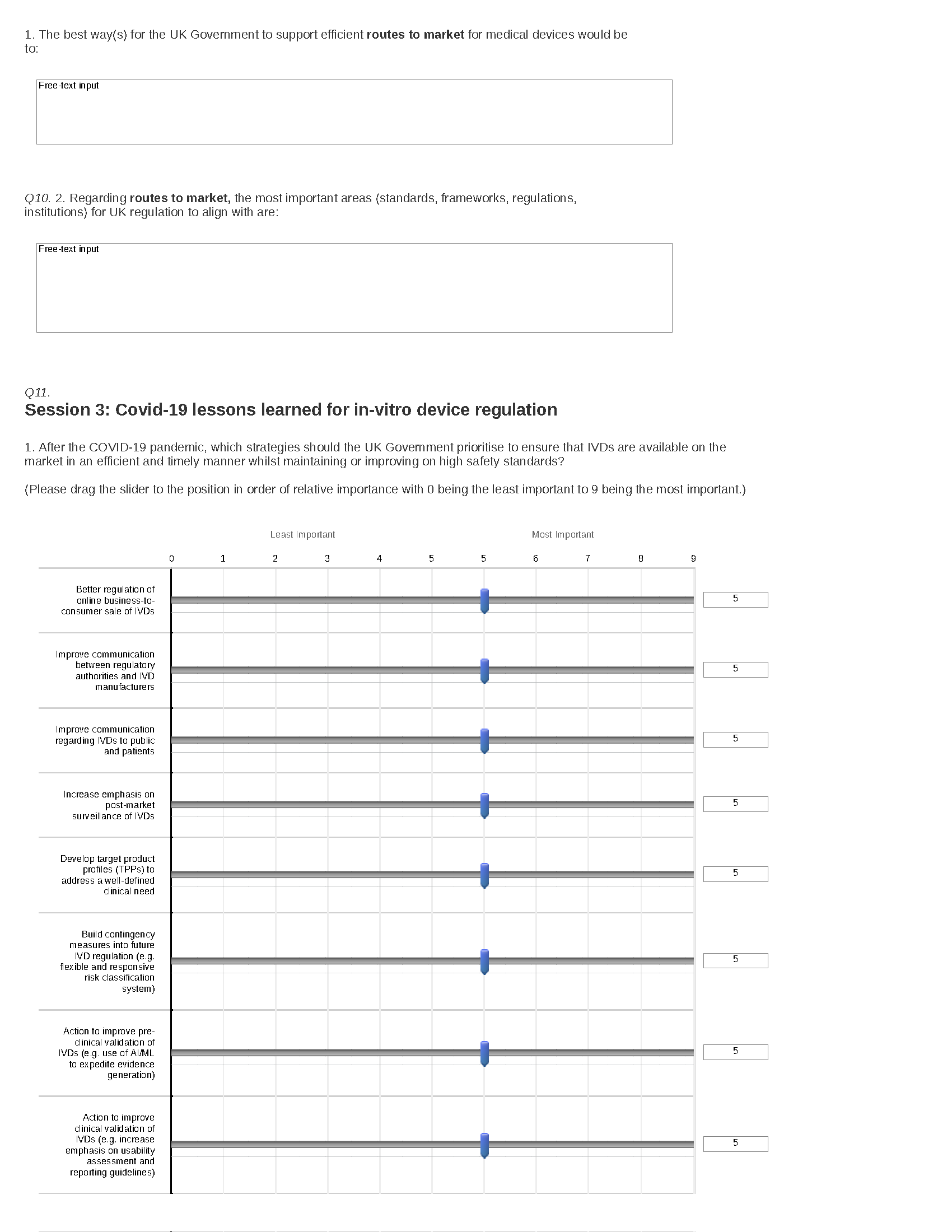

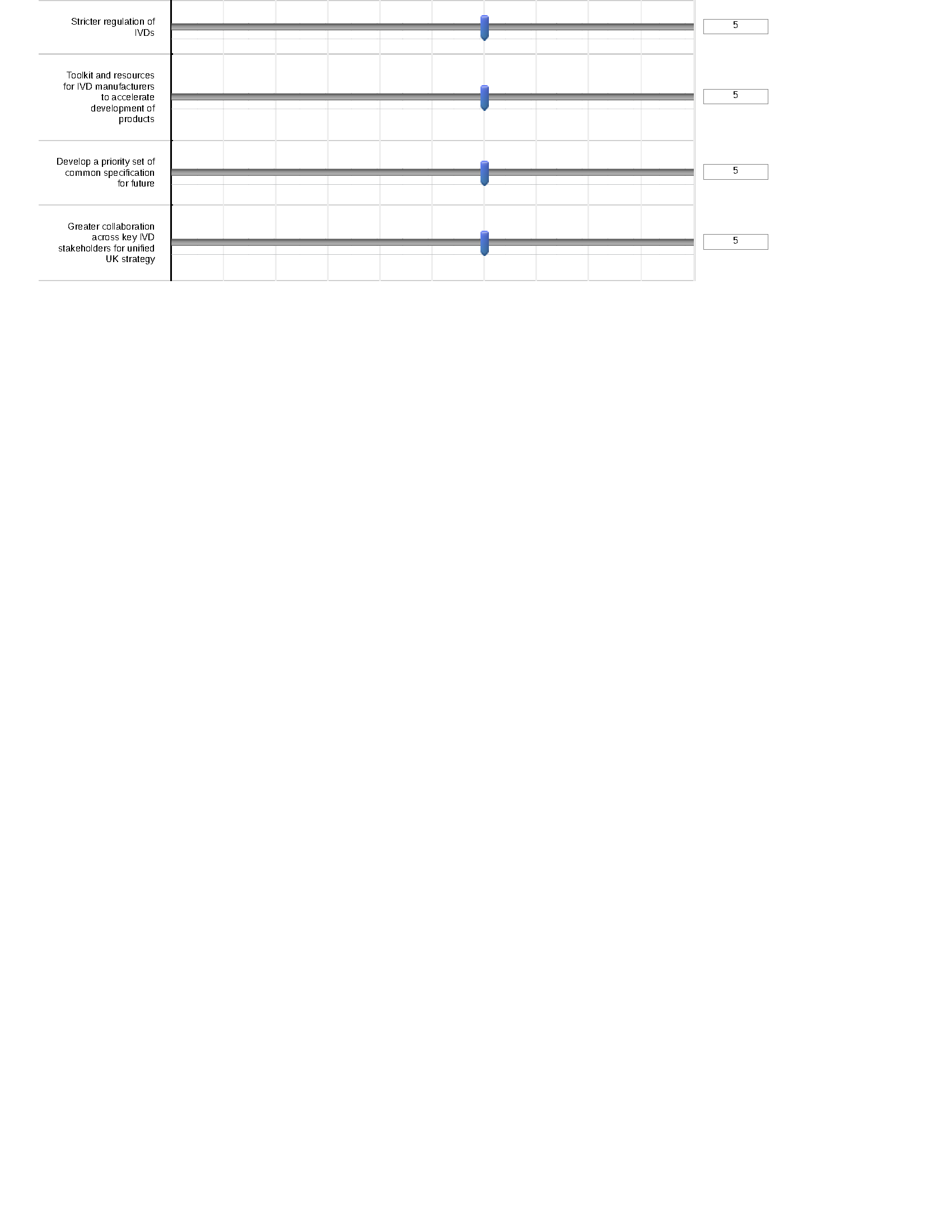


# Appendix 4. Interview Topic Guide

**Introduction:**

- Introduce self to participant
  - *Introduce name/job/role*
- Have you had a chance to read through the participant information sheet?
  - If YES → short summary
    - *Background to study: The aim of this study is to provide robust, multi-stakeholder, cross-sector information that will be used to inform recommendations for the UK Government around possible medical devices regulatory reform that maximises opportunity and minimises risks.*
    - *Nature of interview: We are hoping to discuss the following items in this interview: (a) lessons learnt from Covid-19 regarding in-vitro medical devices regulations; (b) potential alternative routes to the UK market for medical devices; and (c) the transition to a UKCA mark from 1 July 2023. We are happy to be guided by your knowledge/experience and focus the interview on the item(s) that you feel most comfortable/able to discuss. Do you want to talk about any of the items in particular or are you happy to talk about all of them?*
    - *Anonymity/Confidentiality/Withdrawal: The interview will be recorded. All information that is collected about you during the course of the research will be anonymised and kept confidential.  You may withdraw permission for us to use your information without giving a reason up to 5 working days after the interview.*
  - If NO → long summary
    - *Background to study: One of the high-profile, immediate healthcare opportunities afforded by the UK’s decision to leave the EU is reform of regulation around medical devices. Post-Brexit regulatory divergence creates opportunities for both health and economic benefits and also risks. There is an urgent need for robust, multi-stakeholder, cross-sector recommendations around future medical devices regulatory reform that maximise opportunities and minimise risks. The overall purpose of this independent project is to produce such recommendations for the UK Government.*
    - *Nature of interview: We are hoping to discuss the following items in this interview: (a) lessons learnt from Covid-19 regarding in-vitro medical devices regulations; (b) potential alternative routes to the UK market for medical devices; and (c) the transition to a UKCA mark from 1 July 2023. We are happy to be guided by your knowledge/experience and focus the interview on the item(s) that you feel most comfortable/able to discuss. Do you want to talk about any of the items in particular or are you happy to talk about all of them? You can choose to answer the questions in as little or as much detail as you feel comfortable. Feel free to ask to skip questions or ask to return to questions later in the conversation. The interview will last approximately 1 hour.*
    - *Anonymity/Confidentiality/Withdrawal: Please note that the interview will be recorded using an encryption device. The recordings will be removed from the recording device as soon as they are uploaded to a secure, password-protected encrypted University server. The recording will be used to generate an anonymised transcript to facilitate subsequent analysis. All information that is collected about you during the course of the research will be anonymised and kept confidential. Data will be securely stored on the protected University of Birmingham server for 10 years as anonymised linked data but will not be shared outside the research team. All data will be destroyed at the end of 10 years. You may withdraw permission for us to use your information without giving a reason up to 5 working days after the interview.*
- Provide participant with opportunity to ask questions
  - *Do you have any questions you would like to ask us about the background to the study, the nature of the interview, anonymity, confidentiality, or withdrawal?*
- Obtain and record informed consent from participant to proceed with interview
  - *Are you happy for us to proceed with the interview?*

**Interview:**

- **To begin the interview, it’s helpful for us to know a little bit about you.**
  - Can you tell us about what you do?
    - - *Prompt - job(s), role(s)*
- What is your knowledge and/or experience in medical devices?
  - - - *Prompt - length of time, clinical area*
- What is your knowledge and/or experience in regulation around medical devices?
  - - - *Prompt - length of time, organisation*
- **One of the topics we are hoping to discuss with you in today’s interview is routes to (the ways in which they can be made available on) the UK market for medical devices.**
  - Do you know of potential alternative routes to market for medical devices that are currently being used elsewhere/internationally (outside of the EU) that could be used in the UK at the end of its standstill period with the EU?
    - - *Prompt - await RHC, country/region*
- What are the pros and cons of these alternative routes to market for medical devices compared to the routes that are currently being used in the UK?
  - - - *Prompt - costs, timescales, do you perceive there to be any b****lockers/limitations to market currently within the UK market, r****egistering devices, time*
- Reflecting on your answer to the previous question, which of the potential alternative routes to market for medical devices do you think is the most appropriate for the UK and why?
- **Another topic we are hoping to discuss with you in today’s interview is the move to a UKCA mark from 1 July 2023.**
  - What are your thoughts on the implications of an end to the use of EU CE marked medical devices in Great Britain on 1 July 2023?
    - - *Prompt - costs, timescales, supply continuity, change in safety standards, consumer confidence*
- What mitigation work could take place to support industry in the change to the regulatory framework and move to the UKCA mark from 1 July 2023?
  - Why/how would [answer] do that?
- **In-vitro medical devices (IVDs) are devices intended for the *laboratory* testing of samples derived from the human body (*IMDRF definition*). We want to collate the lessons learned from COVID-19 in relation to the application of in-vitro medical devices regulations to provide recommendations for their improvements.**
  - During the COVID-19 pandemic, have any challenges arisen around the application of IVD regulations or use of IVDs?
    - - *Prompt - regulatory approval, partnership with the NHS, UK government and other industry, development of assays*
- Following on from that, are there any ways IVD regulations or approval processes could be changed to overcome these challenges?
  - - - *Prompt - fast-track + flexible route to emergency use authorisation and CE marking, timely information and intelligence from the government/trade association*
- In your opinion, what is the most important lesson you have learned regarding the application of IVD regulations during the COVID-19 pandemic?
- **As we explained at the start, the overall purpose of this project is to produce a set of recommendations around potential future medical devices regulatory reform that maximise opportunities and minimise risks.**
  - What do you think are the potential **opportunities** around future UK regulatory reform around medical devices?
    - - *Prompt - health, economic, life sciences sector*
- What do you think are the potential **risks** around future UK regulatory reform around medical devices?
  - How would you overcome these risks? What do you think is the most appropriate way to overcome these risks?
    - - *Prompt - health, economic, life sciences sector*
- The next 3 questions are about **regulatory** changes the UK could make to encourage international investment, innovation, and safety.
  - How can the UK encourage **international investment** in the medical devices area through **regulatory** changes?
    - Why/how would [answer] do that?
  - How can the UK encourage **innovation** in the medical devices area through **regulatory** changes?
    - Why/how would [answer] do that?
  - How can the UK improve **safety** in the medical devices area through **regulatory** changes?
    - Why/how would [answer] do that? - intentionally, meaningful,
- The next 3 questions are about **non-regulatory** changes the UK could make to encourage international investment, innovation, and safety.
  - How can the UK encourage **international** **investment** in the medical devices area through **non-regulatory** changes?
    - Why/how would [answer] do that?
  - How can the UK encourage **innovation** in the medical devices area through **non-regulatory** changes?
    - Why/how would [answer] do that?
  - How can the UK improve **safety** in the medical devices area through **non-regulatory** changes?
    - Why/how would [answer] do that?
- Is there **anything else** you think is relevant to UK regulatory reform around medical devices that we haven’t spoken about yet?

**Conclusion:**

- Inform participant that interview is over
  - *The interview is now over.*
- Provide participant with opportunity to ask questions
  - *Do you have any questions you would like to ask us at this point in time?*
- Remind participant about anonymity, confidentiality, and withdrawal
  - *As we explained at the start, the interview was recorded. The recording will be used to generate an anonymised transcript to facilitate subsequent analysis and then be destroyed. All information that is collected about you during the course of the research will be anonymised and kept confidential. You may withdraw permission for us to use your information without giving a reason up to 5 working days after the interview.*
- Provide participant with information about next steps including consensus meeting
  - *Moving forwards, we would like to invite you to take part in a consensus meeting with other members of our research team and the other experts we have interviewed. The aim of the consensus meeting is to reach consensus on a set of recommendations for future regulatory reform around medical devices. Any contentious issues identified after analysis of the interviews will be discussed and debated. The consensus meeting will be conducted online via video call on 09 February 2021. Ideally you would attend both the interview and the consensus meeting. If, however, for whatever reason, you are unable to take part in the consensus meeting, that is fine. Are you happy for us to send you an invite to participate in the consensus meeting?*
- Thank participant for taking part
  - *Thank you for participating in this interview.*

# Appendix 5. Reference list of literature included in the rapid review for the theme: *Key implications of the transition to the UKCA or CE UKNI mark*

1. Stanley A. A new era for the dental industry. BDJ Pract. 2020;33(4):18-19. doi:10/ghzh9m

2. Blog: UKCA – What does it mean for medical device companies? Hardian Health. Accessed February 5, 2021. https://hardianhealth.com/blog/2020/9/3/blogukca

3. Brexit and the CE Mark - implications for European regulation of medical devices. Brandwood CKC. Published June 26, 2016. Accessed February 5, 2021. https://brandwoodckc.com/brexit-and-the-ce-mark-implications-for-european-regulation-of-medical-devices/

4. Brexit and the Medical Devices Regulation – Opportunities and Risks. Accessed February 5, 2021. https://www.criticalsoftware.com/news/brexit-and-the-medical-devices-regulation

5. Denissen T. BREXIT impact. Accessed February 5, 2021. https://www.medical-risk.com/regulatory-insights/item/83-brexit-and-the-impact-on-your-business-in-the-eu

6. Brexit Medical Device Implications. Acorn Regulatory. Published August 25, 2016. Accessed February 5, 2021. https://acornregulatory.com/brexit-medical-device/

7. Brexit, medical devices and transfer of notified bodies. What will be the procedure? Accessed February 5, 2021. https://www.engage.hoganlovells.com/knowledgeservices/news/brexit-medical-devices-and-transfer-of-notified-bodies-what-will-be-the-procedure

8. Mitchell P. “Brexit” stuns UK biotech into waiting game, but not all signals are red. Nat Biotechnol. 2016;34(8):787-788. doi:10/ghzh9n

9. Cygan A. De-Europeanisation of UK regulatory governance and the future UK–EU trade relationship. ERA Forum. 2020;20(4):509-529. doi:10/ghzh9s

10. Hervey T, Antova I, Flear ML, McHale JV, Speakman E, Wood M. Health “Brexternalities”: The Brexit Effect on Health and Health Care outside the United Kingdom. J Health Polit Policy Law. Published online October 14, 2020. doi:10/ghzh9q

11. McHale JV. Health law, Brexit and medical devices: A question of legal regulation and patient safety. Med Law Int. 2018;18(2-3):195-215. doi:10.1177/0968533218807255

12. Harris M. Hi-tech firms seek clarity amid Brexit confusion. Phys World. 2019;32(3):10-11. doi:10/ghzh9r

13. Matters B. How leaving the EU might impact the UK medical device industry. Business Matters. Published March 2, 2020. Accessed February 5, 2021. https://bmmagazine.co.uk/in-business/how-leaving-the-eu-might-impact-the-uk-medical-device-industry/

14. Dayan M, Trust N. How will our future relationship with the EU shape the NHS? :36.

15. How will the UK regulate medical device market after Brexit? Accessed February 5, 2021. https://www.medicaldevice-network.com/comment/uk-medical-devices-brexit/

16. Impact of Brexit on UK and EU based Medical Device Manufacturers. Med-Di-Dia: Medical, Digital Health, Diagnostics. Accessed February 5, 2021. https://med-di-dia.com/news/impact-of-brexit-on-uk-and-eu-based-medical-device

17. Impact of Brexit: Medical Devices and CE Marking. Accessed February 5, 2021. https://www.imeche.org/policy-and-press/reports/detail/impact-of-brexit-medical-devices-and-ce-marking

18. Medical Device - Poll # 11 – Implementation of the new EU Medical Device Regulation. Accessed February 5, 2021. https://survey.alchemer.eu/s3/90298955/Medical-Device-Poll-11-Implementation-of-the-new-EU-Medical-Device-Regulation

19. Keogh K, McConaghie G. Medical device use post Brexit: should we be concerned? Bull R Coll Surg Engl. 2017;99(7):250-251. doi:10/ghzh88

20. Medicines and medical devices regulation post-Brexit. Accessed February 5, 2021. https://www.taylorwessing.com/synapse/september16.html

21. MedTech Europe Position Paper on Article 50 Negotiations between the European Union and the United Kingdom (Brexit). Accessed February 5, 2021. https://www.abhi.org.uk/media/1701/mte-brexit-position-paper.pdf

22. Parvizi N, Parvizi S. New Health Technologies: A UK Perspective; Comment on “Providing Value to New Health Technology: The Early Contribution of Entrepreneurs, Investors, and Regulatory Agencies.” Int J Health Policy Manag. 2017;6(12):721-722. doi:10/ghzh9p

23. Position on the implications of the UK exit from the European Union. AESGP. Published March 21, 2019. Accessed February 5, 2021. https://aesgp.eu/articles/aesgp-position-on-the-implications-of-the-uk-exit-from-the-european-union

24. Piorkowska M, Goh V, Booth TC. Post Brexit: challenges and opportunities for radiology beyond the European Union. Br J Radiol. 2017;90(1072):20160852. doi:10/f9tbjm

25. Product safety assessment marks after Brexit – out with the old, in with the new. Accessed February 5, 2021. https://www.penningtonslaw.com/news-publications/latest-news/2020/product-safety-assessment-marks-after-brexit-out-with-the-old-in-with-the-new

26. The European Union Medical Device Regulations: lost in the wash? | Bone & Joint 360. Accessed February 5, 2021. https://online.boneandjoint.org.uk/doi/abs/10.1302/2048-0105.85.360727

27. The immediate futures of health law after Brexit: Law, ‘a-legality’ and uncertainty - Tamara Hervey, Elizabeth M Speakman, 2018. Accessed February 5, 2021. https://journals.sagepub.com/doi/abs/10.1177/0968533218810746

28. Lewis M, Shah BL-O, Ranson P, Manoussakis S. The Impact of Brexit on Medical Devices Regulation in the United Kingdom | Lexology. Accessed February 5, 2021. https://www.lexology.com/library/detail.aspx?g=9483d187-2e01-4df4-ba49-c978f89d16d1

29. The ripple effect of Brexit on medical device regulation. ARC Regulatory. Published September 11, 2020. Accessed February 5, 2021. https://www.arc-regulatory.co.uk/the-ripple-effect-of-brexit-on-medical-device-regulation/

30. UK Medical Device Manufacturers Prepare for Post-Brexit Regulations. Accessed February 5, 2021. <https://www.assemblymag.com/articles/96011-uk-medical-device-manufacturers-prepare-for-post-brexit-regulations?v=preview>

31. Dayan M, Fahy N, Hervey T, McCarey M, Jarman H, Greer S. Understanding the impact of Brexit on health in the UK. :58.

# Appendix 6. Reference list of literature included in the rapid review for the theme: *Evaluating alternative regulatory frameworks or regulatory components for potential adoption by the UK*

1. Fry BM. A Reasoned Proposition To A Perilous Problem: Creating A Government Agency To Remedy The Emphatic Failure Of Notified Bodies In The Medical Device Industry. Willamette J Int Law Dispute Resolut. 2014;22(1):161-198.

2. Anchin U.S. Medtech Challenges at Home and Abroad. Accessed February 5, 2021. https://www.anchin.com/uploads/1406/doc/Pub_2012_RD-LifeSci_MDDI_MedTech.pdf

3. Fiedler BA, Ferguson M. Chapter 2 - Overview of Medical Device Clinical Trials. In: Fiedler BA, ed. Managing Medical Devices Within a Regulatory Framework. Elsevier; 2017:17-32. doi:10.1016/B978-0-12-804179-6.00002-2

4. Fraser AG, Daubert J-C, Van de Werf F, et al. Clinical evaluation of cardiovascular devices: principles, problems, and proposals for European regulatory reform: Report of a policy conference of the European Society of Cardiology†. Eur Heart J. 2011;32(13):1673-1686. doi:10/d4tm6f

5. Rish T. Complete Guide to Bringing a Medical Device to Market. Accessed February 5, 2021. https://www.greenlight.guru/blog/bringing-medical-device-to-market

6. Michie S, Yardley L, West R, Patrick K, Greaves F. Developing and Evaluating Digital Interventions to Promote Behavior Change in Health and Health Care: Recommendations Resulting From an International Workshop. J Med Internet Res. 2017;19(6):e232. doi:10/gbkz7p

7. Van Norman GA. Drugs and Devices: Comparison of European and U.S. Approval Processes. JACC Basic Transl Sci. 2016;1(5):399-412. doi:10/ghkxr2

8. Van Norman GA. Drugs, Devices, and the FDA: Part 2: An Overview of Approval Processes: FDA Approval of Medical Devices. JACC Basic Transl Sci. 2016;1(4):277-287. doi:10/gf7k6w

9. Kramer DB, Tan YT, Sato C, Kesselheim AS. Ensuring Medical Device Effectiveness and Safety: A Cross - National Comparison of Approaches to Regulation. Food Drug Law J. 2014;69(1):1-i.

10. Sorenson C, Drummond M. Improving medical device regulation: the United States and Europe in perspective. Milbank Q. 2014;92(1):114-150. doi:10/f5v467

11. Thompson M, Heneghan C, Billingsley M, Cohen D. Medical device recalls and transparency in the UK. BMJ. 2011;342:d2973. doi:10/bxqtn8

12. Chai JY. Medical device regulation in the United States and the European Union: a comparative study. Food Drug Law J. 2000;55(1):57-80.

13. Santos ICT, Gazelle GS, Rocha LA, Tavares JMRS. Medical device specificities: opportunities for a dedicated product development methodology. Expert Rev Med Devices. 2012;9(3):299-311. doi:10/ghzh75

14. Kahol D, Haycock L, Emich H. Navigating the Maze – Market Access for Medical Devices: Planning Beyond Regulatory Approval. Published online 2015:8.

15. Roche N, Scheuch G, Pritchard JN, et al. Patient Focus and Regulatory Considerations for Inhalation Device Design: Report from the 2015 IPAC-RS/ISAM Workshop. J Aerosol Med Pulm Drug Deliv. 2017;30(1):1-13. doi:10/f9pzqh

16. Bardram JE, Mihailidis A, Wan D, eds. Pervasive Computing in Healthcare. CRC Press; 2007.

17. Sujan MA, Koornneef F, Chozos N, Pozzi S, Kelly T. Safety cases for medical devices and health information technology: involving health-care organisations in the assurance of safety. Health Informatics J. 2013;19(3):165-182. doi:10/f5dx6b

18. SGS Medical Devices Audit, Certification & Training Services. Accessed February 5, 2021. https://www.sgs.co.uk/-/media/local/uk/documents/brochures/sgs-ssc-medical-devices-brochure-a4-en-11-v1.pdf

19. Substantially Unequivalent. Illinois Law Review. Accessed February 5, 2021. https://illinoislawreview.org/print/volume-2014-issue-4/note-substantially-unequivalent-reforming-fda-regulation-of-medical-devices/

20. Mangir N, Roman S, MacNeil S. The changing regulatory landscape for biomedical implants and its relationship to withdrawal of some vaginal mesh products. Curr Opin Urol. 2019;29(4):414-418. doi:10/ghzh78

21. Storz-Pfennig P, Schmedders M, Dettloff M. Trials are needed before new devices are used in routine practice in Europe. BMJ. 2013;346:f1646. doi:10/ghzh77

22. Camp J, Graboyes RF. US Medical Devices: Choices and Consequences. SSRN Electron J. Published online 2018. doi:10/ghzh8h

23. Longley D. Who is Calling the Piper? Is There a Tune? The New Regulatory Systems for Medical Devices in the United Kingdom and Canada. Med Law Int. 1998;3(4):319-345. doi:10/b8k5cq

# Appendix 7. Post-survey Questionnaire Results


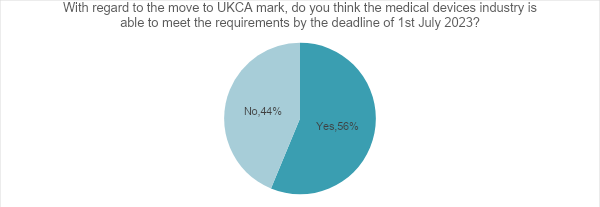


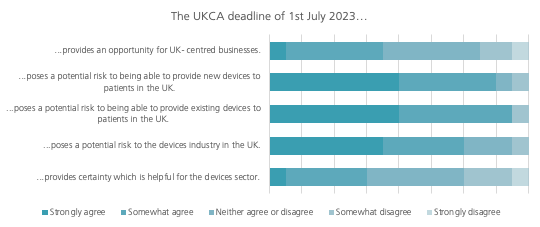


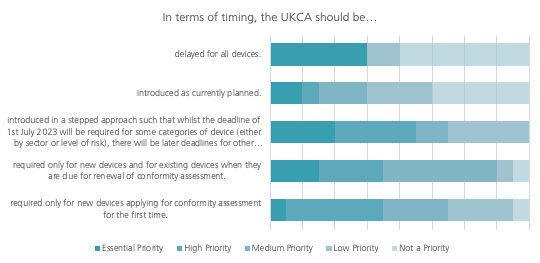

Supplement: Supplementary file 1 — Supplementary file1 (DOCX 425 kb) [file 43441_2022_394_MOESM1_ESM.docx]
